# Comparative Analysis of Electric Field Strength, Magnetic Field Strength and Power Density around the Cell Phone Towers of Varying Characteristics with a Proposed Classification Facilitating Research on Human Population

**DOI:** 10.3390/ijerph192114157

**Published:** 2022-10-29

**Authors:** John Baliah, Balanehru Subramanian, David Livingstone, Bushra Kanwal, Mahmud Uz Zaman, Kumar Chandan Srivastava, Huda Abutayyem, Khalid Al-Johani, Anju P. David, Deepti Shrivastava, Mohammad Khursheed Alam

**Affiliations:** 1 Department of Oral Medicine and Radiology, Indira Gandhi Institute of Dental Sciences, Sri Balaji Vidyapeeth (Deemed to be University), Mahatma Gandhi Medical College and Research Institute Campus, Pondicherry 607402, India; 2 Central Inter-Disciplinary Research Facility, Sri Balaji Vidyapeeth (Deemed to be University), Mahatma Gandhi Medical College and Research Institute Campus, Pondicherry 607402, India; 3 Department of Prosthodontics and Crown & Bridge, Indira Gandhi Institute of Dental Sciences, Sri Balaji Vidyapeeth (Deemed to be University), Mahatma Gandhi Medical College and Research Institute Campus, Pondicherry 607402, India; 4 Independent Researcher, Banisar, Al Baha 65511, Saudi Arabia; 5 Department of Oral and Maxillofacial Surgery and Diagnostic Sciences, College of Dentistry, Prince Sattam Bin Abdullaziz University, Ad Dilam Rd, Ar Rashidiyah, Al-Kharj 16245, Saudi Arabia; 6 Department of Oral and Maxillofacial Surgery and Diagnostic Sciences, College of Dentistry, Jouf University, Sakaka 72345, Saudi Arabia; 7 Department of Clinical Sciences, Center of Medical and Bio-Allied Health Sciences Research, College of Dentistry, Ajman University, Ajman 346, United Arab Emirates; 8 Department of Oral Diagnostic Sciences, Faculty of Dentistry, King Abdulaziz University, Jeddah 21589, Saudi Arabia; 9 Al Jouf Specialist Dental Centre, Sakaka under Ministry of Health, Sakaka 72345, Saudi Arabia; 10 Department of Preventive Dentistry, College of Dentistry, Jouf University, Sakaka 72345, Saudi Arabia; 11 Center for Transdisciplinary Research (CFTR), Saveetha Dental College, Saveetha Institute of Medical and Technical Sciences, Saveetha University, Chennai 602105, India; 12 Department of Public Health, Faculty of Allied Health Sciences, Daffodil lnternational University, Dhaka 1341, Bangladesh

**Keywords:** public health, electric field, electromagnetic field, magnetic field, radiation, radio waves, radiation detection, exposure evaluation, measurements

## Abstract

The continuous exposure of electromagnetic field (EMF) radiation from cell phone towers may possibly have an influence on public health. Each cell phone tower is unique in terms of number of antennas and its associated attributes; thus, the radiation exposure varies from one tower to another. Hence, a standardized method for quantifying the exposure is beneficial while studying the effects of radiation on the human population residing around the cell phone towers. A mere collection of data or human samples without understanding the cell phone tower differences may show study results such as an increase or decrease in biological parameters. Those changes may not be due to the effects of EMF radiation from cell phone towers but could be due to any other cause. Therefore, a comparative study was designed with the aim of quantifying and comparing the electric field strength (EF), magnetic field strength (MF) and power density (PD) on four sides of cell phone towers with varying numbers of antennas at 50 m and 100 m. Further, an attempt was made to develop a PD-based classification for facilitating research involving human biological samples. Through convenience sampling, sixteen cell phone towers were selected. With the use of coordinates, the geographic mapping of selected towers was performed to measure the distance between the towers. Based on the number of antennas, the cell phone towers were categorized into four groups which are described as group I with 1–5 antennas, group II comprising of 6–10 antennas, group III consisting of 11–15 antennas and group IV comprised of towers clustered with more than 15 antennas. The study parameters, namely the EF, MF and PD, were recorded on all four sides of the cell phone towers at 50 m and 100 m. One-way ANOVA was performed to compare the study parameters among study groups and different sides using the Statistical Package for the Social Sciences (SPSS) version 25.0. The mean MF in Group IV was 2221.288 ± 884.885 μA/m and 1616.913 ± 745.039 μA/m at 50 m and 100 m respectively. The mean PD in Group IV at 50 m was 0.129 ± 0.094 μW/cm^2^ and 0.072 ± 0.061 μW/cm^2^ at 100 m. There was a statistically significant (*p* < 0.05) increase in the MF and PD at 50 m compared to 100 m among cell phone tower clusters with more than 15 antennas (Group IV). On the other hand, a non-significant increase in EF was observed at 50 m compared to 100 m in Group II and IV. The EF, MF and PD on all four sides around cell phone towers are not consistent with distance at 50 m and 100 m due to variation in the number of antennas. Accordingly, a PD-based classification was developed as low, medium and high for conducting research involving any biological sample based on quantile. The low PD corresponds to 0.001–0.029, medium to 0.03–0.099 and high to 0.1–0.355 (μW/cm^2^). The PD-based classification is a preferred method over the sole criteria of distance for conducting human research as it measures the true effects of EMF radiation from the cell phone towers.

## 1. Introduction

Electromagnetic field (EMF) radiation is ubiquitous. The electromagnetic spectrum can be divided into ionizing and non-ionizing radiation [1]. In addition to the man-made EMF radiation from cell phone towers, background radiation from natural sources contributes to the average dose received by the general public. In this respect, the situation for EMF radiation is similar to that of ionizing radiation. According to the United Nations Scientific Committee on the Effects of Atomic Radiation Estimates, the global average annual dose from natural background sources was 2.4 and 0.48 mSv from terrestrial sources [2]. In the coastal belt of the Arabian Sea at Kerala, background radiation levels are 20 times higher than the global average effective dose [3]. The radiofrequency (RF) waves used in wireless communication are non-ionizing as they do not ionize biological matter [4]. In recent times, the use of mobile phones has increased tremendously. Consequently, there has been an apparent increase in the number of cell phone towers by various network providers. This, as well as the continuous whole-body nature of continuous exposure, may constitute a public health risk [5], which still requires a scientific assessment.

When a radiofrequency current is supplied to an antenna, the radiofrequency EMF propagates through space with a frequency of 100 kHz to 300 GHz. In particular, precise information regarding the mechanism of the biological effects of RF-EMF radiation has not yet been elucidated. The continuous exposure to EMF from cell phone towers may have acute thermal and chronic non-thermal adverse effects similar to EMF from cell phones. Every interaction between radiofrequency fields and living tissues causes an energy transfer resulting in a temperature rise [6]. Increased body temperature is stabilized and alleviated by blood circulation. Although non-thermal effects do not raise the body temperature sufficiently to impair the structure of tissues, their effects can still be seen as an increase in free radical production in tissues [7]. 

Most animal studies have found no evidence of in vitro RF-induced genetic damage at non-thermal exposure regimes [7,8,9]. Although animal studies can provide qualitative information about possible effects, the findings cannot be extended to generate a credible estimate of human risk.

A literature search revealed that people residing around cell phone towers show statistically meaningful changes with respect to their oral health. Augner et al. (2010) observed an increase in salivary cortisol, alpha-amylase and IgA in high exposure conditions from base transceiver stations [10]. Singh K et al. (2016) observed a decrease in stimulated salivary flow rate among the people residing near base transceiver stations [11]. Thamilselvan S et al. conducted a cross-sectional study to detect the presence of micronuclei in the buccal epithelial cells among a population residing within 10–25 m around three cell phone towers in Chennai, India. The results of the study show that 55% of the population had the presence of micronuclei in at least one field. In the age group under 10 years, 47.1% showed the presence of micronuclei [12]. However, the subject included in the study was based on distance and the means of measuring the distance was not clearly defined. Further, the variation in the number and the type of antenna was also not considered before saliva collection and only four cell phone towers were included in the study. 

Since there are two different schools of thought concerning the safety of EMF radiation from cell phone towers, it is prudent to arrive at a standard method for conducting research in human populations residing near and around cell phone towers, especially involving biological samples.

Hence, this study was designed with the research question, ‘is there a difference in the EF, MF, and PD around cell phone towers on four sides with 1–5 antennas, 6–10 antennas, 11–15 antennas and clusters of towers at 50 m and 100 m?’

The primary aim of the study was to quantify and compare the EF, MF, and PD on four sides around cell phone towers with varying numbers of antennas at 50 m and 100 m. Further, an attempt was made to develop a PD-based classification which can be used to facilitate scientific research involving human biological samples.

## 2. Materials and Methods

### 2.1. Study Characteristics

After obtaining the institutional doctoral and ethical committee clearance of Sri Balaji Vidyapeeth (IEC No. PhD/2016/03/02), the current observational study was conducted in Pondicherry, India. The study was conducted in an urban setup around residential houses with a maximum of two to three floors with background radiation. All the cell phone towers included in the study were roof mounted, containing disk and sector antennas at different positions set by the service provider. 

### 2.2. Sample Characteristics

A total of 16 cell phone towers in Puducherry, India, were selected using convenience sampling. The sample size was calculated by the following Equation (1).

(1) $$\mathrm{Sample}\;\mathrm{size}=n\geq \left(1+\sqrt{g-1\;}\right)\frac{{\left(Z_{1-\alpha /2}+(Z_{1-\beta }\right)}^{2}}{d^{3}}+\frac{Z^{2}{}_{1-\alpha /2}\sqrt{g-1\;}}{2\left(1+\sqrt{g-1\;}\right)}$$

where α was the Type 1 error rate, *β* was the Type 2 error rate, *d* was the expected effect size, *g* was the number of groups to compare and *Z* was the value from the standard normal distribution reflecting the confidence interval, *Z* = 1.96 for 95% confidence. 

### 2.3. Study Protocol

**Figure 1:**
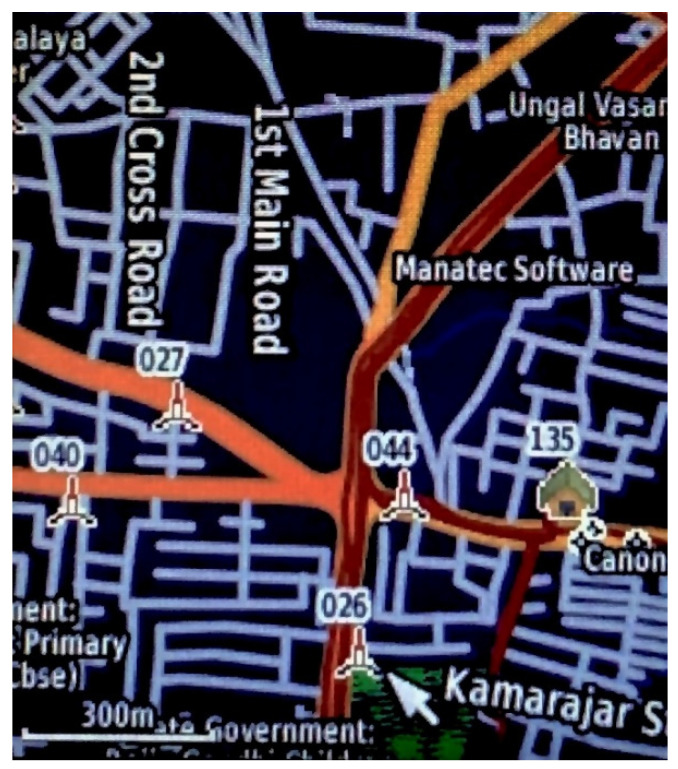
Geographical mapping of cell phone towers using Garmin etrex 20× device. The short white arrow indicates the cell phone tower.

Based on their GPS coordinates, the geographical mapping of the selected 16 cell phone towers was performed in order to calculate the distance between each tower (Figure 1). 

**Figure 2:**
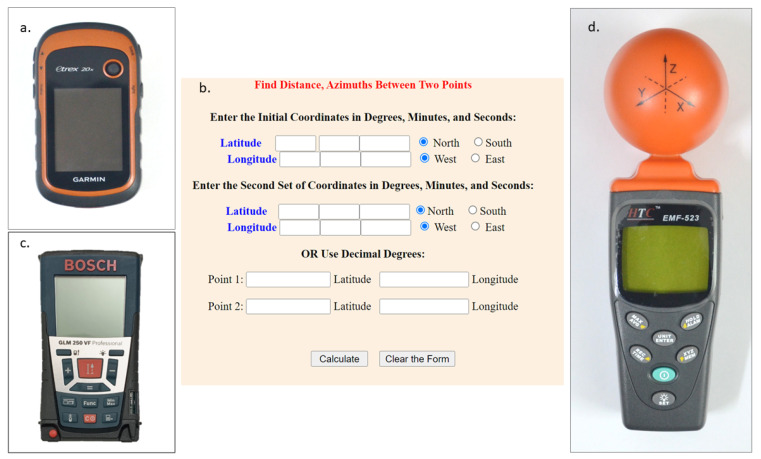
(**a**) Garmin etrex 20x device to identify the cell phone tower coordinates; (**b**) FCC Online conversion tool for calculating distance between two cell phone towers based on two coordinates; (**c**) Bosch GLM 250 VF Professional range finder to measure the distance around the cell phone tower; (**d**) HTC EMF-523 to measure EF, MF and PD.

For geographical mapping of cell phone towers, a Garmin etrex 20x device (Garmin, New Taipei City, Taiwan, China) was used. The device provides the global positioning system (GPS), global navigation satellite system (GLONASS) and wide area augmentation system (WAAS) and a greater accuracy of ±3 m (Figure 2a). Using the two sets of cell phone tower coordinates in the Online Federal Communication Commission tool (FCC), the distance between cell phone towers was calculated and found to be more than 200 m (Figure 2b) [13]. 

Later, based on the number of antennas, the cell phone towers were categorized into four groups, where group I consisted of towers with 1–5 antennas, group II had towers with 6–10 antennas, towers with 11–15 antennas are considered in group III, and lastly, tower clusters with more than 15 antennas were in group IV. Cell phone towers with both disk and sector antennas were included in the study. Corresponding data such as the number of disk- and sector-shaped antennas in a cell phone tower in all four groups and their PD range are given in Table 1. The distances, 50 m and 100 m, were measured using the Laser range finder (Bosch Professional GLM 250 VF, Switzerland) directed towards the lowest antenna in the cell phone tower. The measuring range of this tripod-mounted device was 0.05–250 m with a high accuracy of ±1 mm (Figure 2c).

The EF, MF and PD were measured at 50 and 100 m on all four sides around the cell phone tower using the pre-calibrated HTC EMF 532 RF three-axis field strength meter (HTC, Mumbai, India) (Figure 2d). This CE-certified HTC EMF 523 RF meter was designed for measuring and monitoring radiofrequency electromagnetic field strength, precisely calibrated in the frequency range of 50 MHz–3.5 GHz. It used an isotropic triaxial measurement with a sample rate of 3 times per second. The dynamic range of the meter was typically 75 dB. The absolute error at 1 V/m and 2.45 GHz was ±1.0 dB. The device measured the electric filed strength directly and displayed the calculated magnetic field strength and power density. The device was held at one arm’s length at chest height for a minimum of at least two minutes and the maximum average values of EF, MF and PD were measured. Three consecutive measurements were taken and the third value was recorded. Three measurements were taken to ensure that the EF, MF and PD values were genuinely representative of that particular area. All the measurements were taken during the daytime between 9 a.m. and 11 a.m. No other EMF producing devices or sources such as mobile phones and electrical lines were present while recording the data.

The EMF measurement on four sides around the cell phone tower followed the same pattern of measurement, namely, north, east, south and west. The max average values of the EF, MF and PD were recorded at 50 and 100 m on all four sides. 

### 2.4. Statistical Analysis

The data were subjected to statistical analysis. One-way ANOVA was performed to compare the EF, MF, and the PD among the four study groups and different sides using SPSS version 25.0 (SPSS Inc., Chicago, IL, USA). 

## 3. Result

In group IV, a statistically significant (*p* < 0.05) higher mean MF and PD are observed at 50 m compared to 100 m. On the other hand, a statistically non-significant (*p* > 0.05) increase in mean MF and PD are observed at 100 compared to 50 m in Groups II and III. The inter- and intragroup comparative analysis of EF, MF and PD among the study groups at different distances is presented in Table 2. 

**Figure 3:**
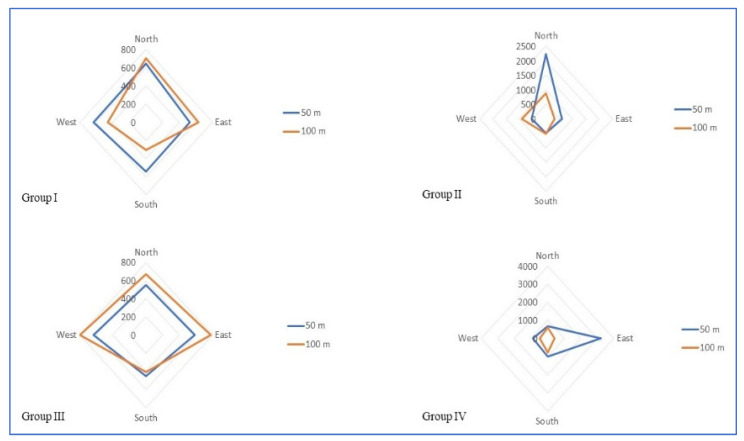
Radar chart showing electric field strength distribution around cell phone towers among different study groups.

**Figure 4:**
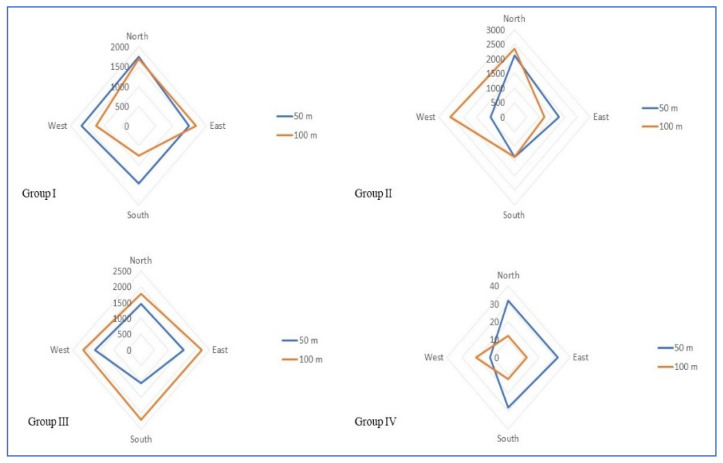
Radar chart showing magnetic field strength distribution around cell phone towers among different study groups.

**Figure 5:**
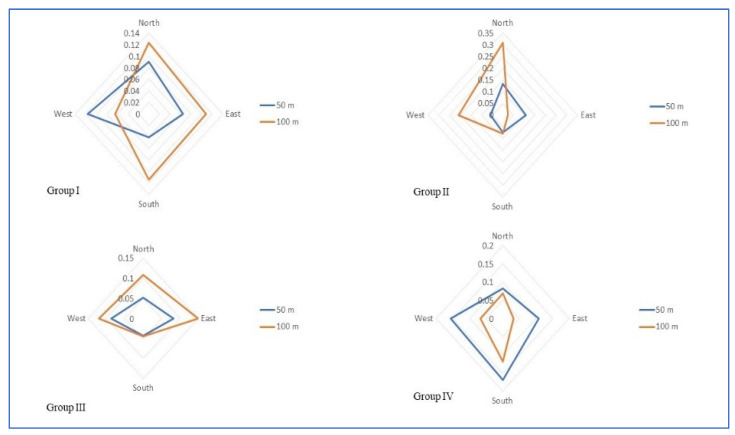
Radar chart showing power density distribution around cell phone towers among different study groups.

The inter- and intragroup comparative analysis of EF, MF and PD among the study groups at different directions is presented in Table 3. Group-wise comparisons of EF (Figure 3), MF (Figure 4) and PD (Figure 5) around the cell phone towers at 50 m and 100 m on all four sides are shown in the radar chart. 

With respect to the EF, MF, and the PD in all four directions around the cell phone tower at 50 and 100 m, a typical bow and tie pattern is observed among all four Groups. On three sides, it is less and on the fourth side, the EF, MF, and PD are quite high, hence the bow and tie pattern. Hence, the EF, MF and PD in all four directions are not consistent with the distance in the present study. Due to this variation in all four sides around the cell phone towers, the distance-based grouping cannot be used in research involving biological samples for this population. Hence, there is a need for a standard alternative reliable method of classification. 

Since the entire dataset with respect to power density from all four groups around cell phone towers is available in the present study, we have developed a new power density-based classification as low, medium and high, based on quantile after excluding the outliers (Table 4). The power density data from the present study are arranged in ascending order from lowest to the highest and divided into three equal parts based on quantiles. To minimize the bias in forming the groups, we opted to use a quantile, as this statistical concept possesses an objective definition and a clear meaning. For categorizing into low, medium and high, the concept of quantiles is ideal to generate ordinal data and it assigns the same number of data values to each class. There are no empty classes or classes with too few or too many values. The drawback of the quantile classification is that features with widely different values can end in the same group, while similar values can be placed adjacent groups. This distortion is minimized by classifying PD into three groups, labelled as low, medium and high, instead of into two groups.

## 4. Discussion

EMF radiation from cell phone towers is a major public health concern among the population residing near and around them [14]. According to the present study, the distance-based grouping may not yield a valid correlation with EMF radiation effects as it is not uniform around the cell phone towers at 50 and 100 m. It exhibited a bow and tie pattern. Several factors contribute to this variation such as type of antennas, the direction of antennas, angulation and operating power.

According to the present study, cell phone towers with a similar number of disk and sector antennas are rare. As shown in Table 1, the range of PD among the different types of antennas, such as disk and sector antennas, at 50 and 100 m is also different. According to Zothansiama et al., despite having the same number of disk and sector antennas (six disk and four sector antennas), a variation of power density may be observed [15]. Hence, this type of antenna-based classification cannot be used for research purpose.

Another relevant parameter, the power of the cell phone tower, usually ranges between 10 and 50 W as demanded by the International Commission of on Non-Ionizing Radiation protection [16]. The data with respect to the input power of the cell phone tower can be important in terms of EF, MF and PD. However, the PD-based classification is practical and easier to apply than a classification based on the power input to the cell phone tower. The attenuation by brick walls and other materials will change the PD regardless of the input power provided to the base station. Hence, PD measurements inside the house are more practical and relevant for conducting epidemiological studies. Hence, PD-based classification is a preferred method rather than input power-based classification.

The power density is chosen for the following reasons. As per the FCC’s RF exposure guidelines, the maximum permissible exposure level to the general public is based on PD, which is 580 μW/cm^2^. This PD is several times greater than the RF value found around the cell phone towers. The FCC-adopted guidelines are identical to those of the National Council on Radiation Protection and IEEE [17]. Hence, we prefer power density over EF and MF.

The following equation relates PD, EF and MF (2):

(2) Pd=E×H

where *Pd* is power density, *E* is electric field strength, and *H* is magnetic field strength. The unit of PD for the above equation is W/m^2^. As 1 mW/cm^2^ has the same power density as 10 W/m^2^, the following equation can be used to obtain these units directly (3):

(3) Pd=0.1×E×H

where *Pd* is power density, *E* is electric field strength, *H* is magnetic field strength and 0.1 is the conversion from 10 W/m^2^ to 1 mW/cm^2^. The unit of PD for the above equation is mW/cm^2^ [18]. In the present study, we prefer μW/cm^2^ in accordance with FCC exposure guidelines.

According to the FCC guidelines for cellular antenna sites, the majority of cell phone towers operate at an Effective Radiated Power (*ERP*) of 100 W or less, which corresponds to a Total Radiated Power (TRP) of 5–10 W [17]. The *ERP* of an antenna is defined as the product of power transmitted to the antenna and antenna gain. It is measured in W. ERP is calculated by the following Equation (4):

(4) ERP=Pt×Ag

where *ERP* is the Effective Radiated Power, *Pt* is the total power transmitted by the antenna and *Ag* is the antenna gain; *Pt* = Radiofrequency power–cable loss [19].

The TRP is usually lower than the ERP due to conductor loss, dielectric loss and unwanted surface wave in the cell phone tower antenna material [20].

In the present study, ERP and TRP were not measured. Rather, PD was measured around the cell phone towers and used in developing a classification.

Power density depends additionally on the angle between EF and MF and their angle to the exposed plane, such as a human participant in medical research. There are two closely placed terms, namely radiation intensity and power density. Power density is the rate of flow of electromagnetic energy or power per unit area. It is expressed as μW/cm^2^.

Radiation intensity is defined as the power per unit solid angle [21]. It is expressed in W/sr. In the present study, we measured the power density and not the radiation intensity.

Ahmad A et al. measured the EMF using a spectrum analyzer at various distances from 25 m to 200 m from selected towers and observed an increase in PD at 25 m compared to 200 m among different network providers [22]. However, the measurements are not taken around the cell phone towers at various distances and differences in the number of antennas are also not considered and thus the results are not comparable to the present study. Kim BC suggested different EMF measurement procedures for in-situ and environmental measurement from operating base stations. Concerning environmental human electromagnetic measurement, the exposure is determined by the maximum value at the highest field position at several places [23]. In the present study, the maximum average value was used for measuring the EF, MF and PD. According to Cooper TG, the EMF radiation around cell phone towers is very low and is 0.002–2% of the ICNIRP general public reference level [24]. Wu et al. in 2013 observed similarly low levels of EMF radiation around cell phone towers [25]. However, a low level alone is not sufficient for research with the biological sample. A classification based on the PD of that particular geographical study area is required for studying human biological samples. To understand the true effects of EMF radiation from cell phone towers, a PD-based classification for that particular geographical area is preferred compared to distance-based grouping. Katerina et al. elaborated on the in vivo and in vitro methodology of examining the possible harmful effects of mobile phone radiation and their associated challenges. The article described the experimental parameters, accurate setting, description of dosimetry, recommendations for the technical parameters of the experiments and defined the source of radiation. Hence, there is a need for a similar standardized method for studying the human population around the cell phone towers [26]. Based on the present study, PD appears to be a reliable parameter and thus PD-based classification is developed for studying the human population. 

Yazan et al. observed that factors such as different building materials restrict the penetration of EMF radiation from cell phone towers reaching inside houses. EMF radiation from different sources inside houses has a proportional effect on blood glutathione S transferase activity compared to the EMF radiation from cell phone towers. The study is based on distance as a legitimate variable for conducting research [27]. In the present study, the PD is not constant with the distance around the cell phone towers, hence distance cannot be considered a legitimate variable. However, PD-based classification needs to be considered to study the health effects of EMF radiation inside the house due to attenuation by the building materials.

Koppel et al. observed the EF differences inside the house and on the balcony in two apartments, one with high and another with low EMF radiation from the cell phone towers [28]. The key information here is to measure the EMF radiation inside the house. According to the present study, PD-based classification is preferred over the EF-based classification as there is no statistical significance with respect to EF at varying differences around the cell phone towers. 

Sultan Ayoub Meo et al. observed a delay in fine and gross motor skills, spatial working memory and attention in school adolescents compared to students who are exposed to low RF-EMF. In School 1, PD was 2.010 μW/cm^2^ and in School 2, PD was 10.021 μW/cm^2^ [29]. In the present study, the highest recorded PD was 0.35 μW/cm^2^. Hence, due to the huge differences in PD values, it is prudent to measure the PD for that particular geographical study area and develop a classification for conducting research on human populations. Further, recording PD inside the house is important in such a population and care should be taken to switch off all the EMF-producing devices inside the house while recording PD.

The feeling of proximity of a cell phone tower by itself could cause the development of symptoms such as a headache [14]. However, such ‘feelings’ are subjective. Such a population can be identified by an interview or questionnaire and health effects can be determined. Even in such studies, PD inside the house is preferred as it could reveal the true picture of EMF radiation regardless of the active antenna types, position of antennas, installation of the antennas (ground based or roof mounted) and power input to the cell phone tower. The measured power density could be compared with the regulatory guidelines of that particular country and disclosed to the study participant. It could also assist in educating the study participant. However, if distance is considered for studying the psychological aspect of an individual with respect to proximity of a cell phone tower, it is not the true effects of EMF radiation from the cell phone tower but rather a subjective perception by the study participant. In such situations, care should be taken in disclosing the correct means of measurement, from where to where, how the measurements are made, device name, manufacturer and the accuracy of the device.

The reported health impacts of EMF radiation from cell phone towers include headaches, sleep disturbances, reduced memory, psychic excitation, nervousness, stress, distress, hunger, lethargy, neurological effects and carcinogenic effects [30].

Zothansiama et al. observed that radiofrequency radiation from cell phone towers increases the frequency of micronuclei in the cultured human peripheral blood lymphocytes among the population residing within a perimeter of 80 m from the cell phone towers. There is also a statistically significant reduction in glutathione, catalase and superoxide dismutase activities in the plasma of exposed individuals. The induction of micronuclei could be due to the increase in free-radical production by EMF radiation [15]. According to this present study, distance is not a reliable parameter for conducting research in a human population residing around cell phone towers.

According to this present study, the PD around the cell phone towers is well within the International Commission on Non-Ionizing Radiation Protection (ICNIRP) safety level of 10 W/m^2^.

### 4.1. Strength and Limitations of the Study

In the present study, EF, MF and PD were measured with a pre-calibrated high-precision three axis meter so that the data obtained were accurate. In addition, the distances of 50 and 100 m around the cell phone tower from the lowest antenna were measured with the high precision range finder device. Further, the high-precision GPS device with greater accuracy was used to measure the cell phone tower coordinates. Hence, the data obtained from the present study are reliable. 

The current study has a few limitations as well. The present research could have included a larger sample size in terms of cell phone towers. The average spatial distribution could be used to identify and differentiate areas with high and low radiation within urban or rural environments. This guides the researcher in selecting the cell phone towers. It was not considered in the present study because no relevant data are available. The input power of the cell phone tower determines the EF, MF and PD and was not considered in this study. The PD-based classification is more workable as it can be readily measured in the place of interest. The quantile method was used for classification into low, medium and high categories.

### 4.2. Future Direction

This classification can be readily used for studying the effects of EMF radiation from cell phone towers on human saliva, serum, plasma, blood, semen or any other samples in the local population. The PD measurement must be performed inside the house and appropriately categorized as low, medium and high as it also includes the attenuation by the walls and roof. The other EMF-producing devices inside the house must also be included and assessed with a validated questionnaire to determine the daily and weekly duration of use. The human biological changes due to 5G technology can also be determined by this method.

## 5. Conclusions

The EF, MF and PD are not consistent at 50 and 100 m on all four sides around cell phone towers due to the variation in the number of antennas. Hence, instead of distance, the PD-based classification is developed as low, medium and high based on quantile. This classification is a preferred method over distance for conducting human research as it would measure the true effects of EMF radiation from cell phone towers. The PD measurement must be performed inside the house prior to sample collection with adequate consideration of other EMF sources which could alter the levels of biological samples.

## Figures and Tables

**Table 1:** Number of disk and sector antennas in a cell phone tower in all four groups and their PD range.

Groups	Cell Phone Tower	Number of Disk Antenna	Number of Sector Antenna	Range of PD at 50 m (μW/cm^2^)	Range of PD at 100 m (μW/cm^2^)
Group I	1	2	3	0.01–0.236	0.002–0.013
	2	0	5	0.01–0.28	0.06–0.157
	3	1	3	0.001–0.025	0.001–0.307
	4	0	4	0.026–0.176	0.008–0.317
Group II	1	0	6	0.052–0.239	0.005–0.328
	2	1	5	0.03–0.327	0.015–0.307
	3	4	5	0.004–0.10	0.02–0.072
	4	3	7	0.002–0.1	0.001–0.71
Group III	1	7	7	0.014–0.032	0.012–0.355
	2	1	10	0.037–0.264	0.05–0.183
	3	4	7	0.019–0.09	0.032–0.084
	4	6	7	0.006–0.18	0.005–0.167
Group IV	1	8	16	0.095–0.322	0.01–0.322
	2	9	11	0.018–0.118	0.021–0.096
	3	9	13	0.116–0.336	0.011–0.078
	4	9	7	0.04–0.155	0.021–0.156

**Table 2:** Inter- and intragroup comparative analysis of electric field strength, magnetic field strength and power density among the study group at different distances.

SG	EF		*p* Value ^‡^	MF		*p* Value ^‡^	PD		*p* Value ^‡^
	50 m	100 m		50 m	100 m		50 m	100 m	
I	588.88 ± 426.87	525.79 ± 458.00	0.690	1590.90 ± 1116.30	1345.17 ± 1166.39	0.547	0.07 ± 0.09	0.10 ± 0.11	0.519
II	962.65 ± 1528.52	657.68 ± 515.25	0.455	1545.02 ± 1083.03	1784.84 ± 1406.43	0.593	0.09 ± 0.08	0.15 ± 0.21	0.283
III	555.19 ± 292.34	661.48 ± 358.62	0.365	1440.84 ± 832.57	2085.10 ± 1157.29	0.081	0.06 ± 0.06	0.10 ± 0.08	0.168
IV	1448.51 ± 2566.34	574.34 ± 316.66	0.186	2221.28 ± 884.85	1616.91 ± 745.03	0.045 *	0.12 ± 0.09	0.07 ± 0.06	0.049 *
*p* value ^†^	0.315	0.754		0.116	0.327		0.190	0.362	

Note: ^†^ One-Way ANOVA; ^‡^ Unpaired *t* Test; * *p* < 0.05; SG—Study Groups; EF—Electric field strength measured in mV/m; MF—Magnetic field strength measured in μA/m; PD—Power density measured in μW/cm^2^; Results are expressed in mean ± standard deviation.

**Table 3:** Inter- and intragroup comparative analysis of Electric field strength, magnetic field strength and power density among the study group at different directions.

Study Groups	Electric Field Strength (EF)				*p* Value	Magnetic Field Strength (MF)				*p* Value	Power Density (PD)				*p* Value
	North	South	East	West		North	South	East	West		North	South	East	West	
I	676.95 ± 470.22	581.06 ± 359.06	424.52 ± 426.37	546.82 ± 524.80	0.732	1722.53 ± 1190.4	1577.47 ± 925.56	1108.45 ± 1083.08	1463.69 ± 1404.32	0.750	0.106 ± 0.11	0.086 ± 0.09	0.077 ± 0.11	0.090 ± 0.11	0.958
II	1551.08 ± 2048.39	463.96 ± 299.98	502.26 ± 476.36	723.36 ± 536.29	0.187	2239.77 ± 955.32	1331.31 ± 978.84	1356.85 ± 1277.38	1731.79 ± 1642.25	0.439	0.220 ± 0.22	0.066 ± 0.07	0.076 ± 0.10	0.132 ± 0.18	0.215
III	609.61 ± 170.58	681.52 ± 510.99	428.91 ± 230.82	713.30 ± 269.52	0.312	1622.88 ± 471.32	1898.16 ± 1333.27	1626.15 ± 1496.50	1904.70 ± 709.08	0.914	0.079 ± 0.049	0.115 ± 0.130	0.043 ± 0.035	0.103 ± 0.069	0.311
IV	651.06 ± 199.95	1816.22 ± 3718.41	893.03 ± 355.12	685.39 ± 427.14	0.574	1736.51 ± 522.08	1555.037 ± 924.40	2386.18 ± 917.10	1998.66 ± 932.94	0.245	0.076 ± 0.042	0.070 ± 0.075	0.143 ± 0.092	0.112 ± 0.102	0.260
*p* Value	0.245	0.464	0.064	0.852		0.467	0.760	0.197	0.831		0.118	0.734	0.185	0.920	

Note: SG—Study Groups; EF—Electric field strength measured in mV/m; MF—Magnetic field strength measured in μA/m; PD—Power density measured in μW/cm^2^; Results are expressed in mean ± standard deviation.

**Table 4:** Proposed classification based on the power density.

Category	Power Density (µW/cm^2^)
Low	0.001–0.029
Medium	0.03–0.099
High	0.1–0.355

## Data Availability

The data is available on reasonable request from the corresponding author.
